# Associations between cardiometabolic index and sleep disorders: Results from NHANES 2015 to 2020

**DOI:** 10.1097/MD.0000000000049295

**Published:** 2026-07-03

**Authors:** Ximeng Peng, Wenbo Wang, Congwenjun Zhang, Hengyue Liu, Yunyi Liu, Dong Wang

**Affiliations:** a School of Medicine, Jianghan University, Wuhan, Hubei, China.

**Keywords:** cardiometabolic index, cross-sectional study, NHANES, sleep disorders

## Abstract

Sleep disorders have already been recognized as a public health concern, with evidence suggesting that lipid metabolism significantly affects sleep quality and circadian rhythms. Cardiometabolic index (CMI), a novel lipid index, has been confirmed to be associated with obesity-associated metabolic disorders, such as diabetes and cardiovascular disorders. Despite these findings, the exact relationship between CMI and sleep disorders remains to be fully validated. Consequently, our objective was to investigate the association between CMI and sleep disorders using data from the National Health and Nutrition Examination Survey (NHANES). Based on data from NHANES spanning 2015 to 2020, this study employed weighted multivariate logistic regression and restricted cubic spline (RCS) regression analysis to investigate the relationship between CMI and sleep disorders. Additionally, receiver operating characteristic analysis and the area under the curve were also utilized to assess the predictive performance of CMI for sleep disorders. Subgroup analyses and interaction tests were conducted to examine the consistency of this association across different populations. A positive association between CMI and sleep disorders was found in a cohort comprising 5817 participants aged 20 and above, which was further verified to be nonlinear through RCS regression analysis. Within the context of a fully adjusted model, when CMI was considered as a continuous variable, each one-unit increase was linked to a 17% increase in the prevalence of sleep disorders (odds ratio [OR] = 1.17; 95% CI: 1.06–1.29; *P* = .004). Upon stratifying CMI into quartiles, it was observed that participants in the highest quartile exhibited an 81% greater risk of developing sleep disorders compared to those in the lowest quartile (OR = 1.81; 95% confidence interval [CI]: 1.29–2.54; *P* = .002). Subgroup analyses and interaction tests demonstrated that the association between CMI and sleep disorders remained consistent across different subgroups, and no significant modification of this relationship was observed by other covariates except for body mass index. An elevated CMI is correlated with a higher probability of sleep disorders among individuals in the United States. Given the difficulty in establishing a causal relationship between the 2, further extensive prospective studies are necessary to investigate the role of CMI in the development of sleep disorders.

## 1. Introduction

Healthy sleep patterns are characterized by optimal sleep duration (7–8 hours), the absence of excessive sleepiness, early chronotype traits (feeling more alert in the morning), no snoring, and no frequent daytime sleepiness.^[1]^ Furthermore, sleep disorders, including insomnia, sleep-related breathing disorders, central hypersomnolence disorders, and other sleep disorders^[2]^ are currently recognized as a public health concern by the Centers for Disease Control.^[3]^ Previous studies have shown that sleep disorders often lead to adverse health outcomes, being significantly associated with decreased quality of life, metabolic disorders,^[4]^ cardiovascular diseases, and increased mortality,^[5]^ and can even result in cognitive decline.^[6]^ In addition, the prevalence of sleep disorders has been on the rise over the past few decades, reaching a relatively high level. For instance, a recent study revealed that up to 27.1% of adults in the United States suffer from sleep disorders, and these disorders are linked to an additional healthcare cost of about $3400 to $5200 per person per year.^[7]^ Given the widespread occurrence of sleep disorders and their association with negative health consequences, it is crucial to investigate the factors that contribute to the development of sleep disorders.

The mechanisms underlying sleep disorders are complex and can be influenced by a wide range of factors, including genetic, psychological, environmental, and biological elements. In recent years, the relationship between metabolic characteristics and sleep has garnered increasing attention from researchers, with substantial evidence suggesting a shared pathophysiological basis between the 2. Studies have found that the prevalence of sleep disorders is often associated with cardiometabolic comorbidities, such as diabetes, impaired lipid metabolism, and metabolic syndrome.^[8]^ Additionally, several new lipid indices, such as the visceral obesity index,^[9,10]^ which integrates waist circumference (WC), body mass index (BMI) and the triglyceride-to-high-density lipoprotein cholesterol (TG/HDL-C) ratio, have been suggested to be associated with obstructive sleep apnea.^[11]^ However, the phenomenon known as the “obesity paradox” associated with BMI has been raised in several studies, indicating limitations in its predictive value for sleep disorders.^[12,13]^ In 2015, Wakabayashi and colleagues modified the lipid accumulation product formula, aiming to more comprehensively reflect an individual’s obesity level and lipid profile, thereby better assessing cardiometabolic risk. They replaced TG with the TG/HDL-C ratio, substituted WC with the waist-to-height ratio (WHtR), and thus developed the cardiometabolic index (CMI).^[14]^ Compared to other lipid metabolism indices, CMI offers a more comprehensive and precise evaluation of cardiometabolic risk. However, the association between CMI and sleep disorders has not been independently studied.

Therefore, the aim of this study is to investigate the relationship between CMI and sleep disorders using cross-sectional data from the National Health and Nutrition Examination Survey (NHANES) database, providing valuable insights for researchers and healthcare professionals.

## 2. Material and methods

### 2.1. Data accession and study population

The cross-sectional data were collected from NHANES, a nationwide study conducted by the National Center for Health Statistics (NCHS), aimed at assessing the nutritional intake and health status of the US population. NHANES utilizes a complex multistage probability sampling technique to provide a sample that accurately represents the entire population of non-institutionalized US residents.^[15]^ Throughout the research, participants completed household interviews, where they provided important demographic, socioeconomic, and health-related information. In addition, participants received physical exams and laboratory testing at certain mobile examination centers. All NHANES research methods were approved by the NCHS Research Ethics Review Board, and informed permission was acquired from each participant. Detailed information about NHANES study designs and data may be found at www.cdc.gov/nchs/nhanes/.

**Figure 1.**
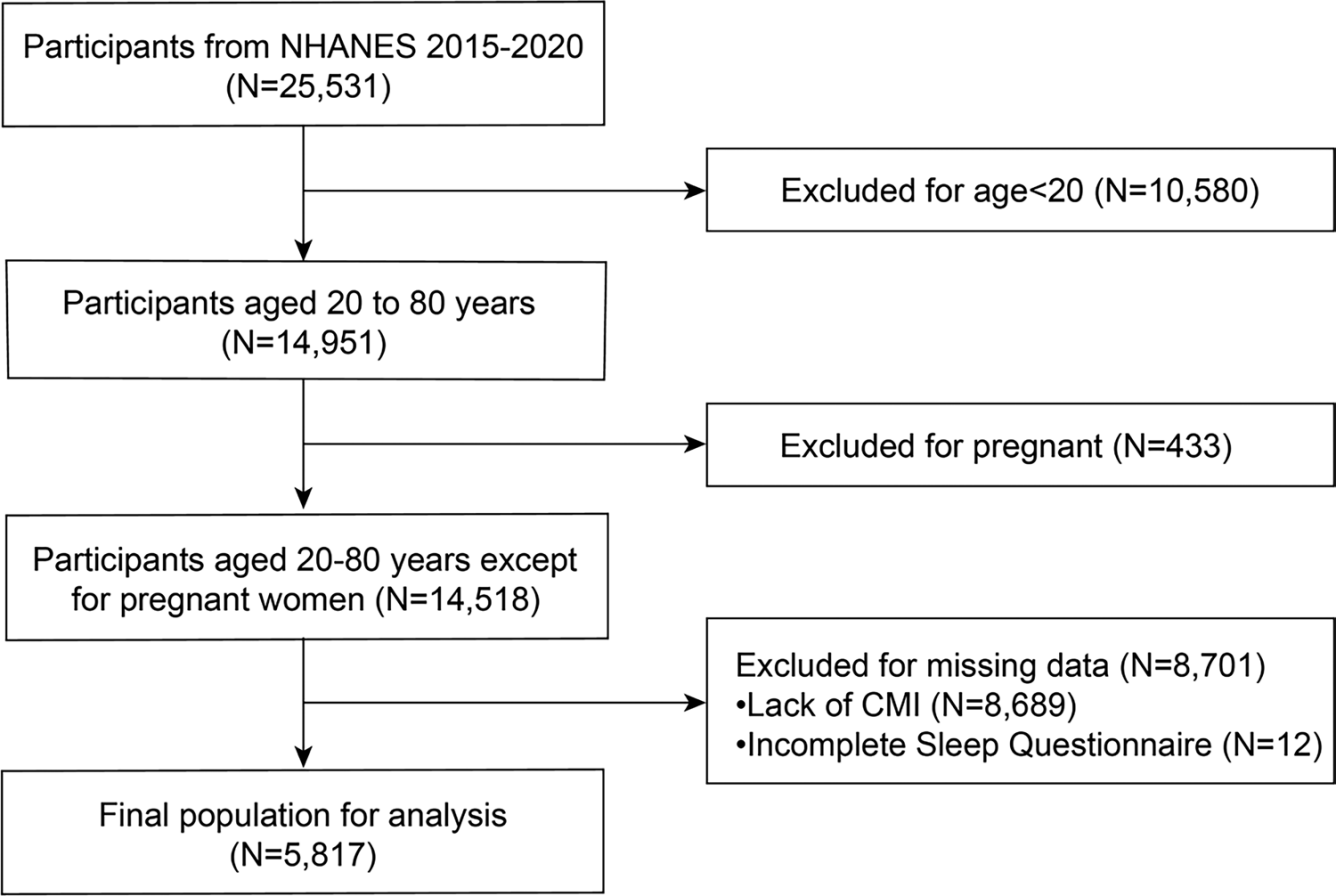
Flow chart of participant selection. CMI = cardiometabolic index, NHANES = National Health and Nutrition Examination Survey.

For this study, we recruited participants from NHANES during 2015 to 2020. Initially, a total of 25,531 individuals were enrolled in the study. After excluding participants who were under 20 years of age (n = 10,580), those who were pregnant (n = 433), and cases with missing data on CMI (n = 8689) and sleep disorders (n = 12), our final analysis included 5817 eligible participants (Fig. 1).

### 2.2. Cardiometabolic index

As reported by Wakabayashi and Daimon,^[16]^ CMI was calculated as the product of WHtR and the TG/HDL-C ratio, representing anthropometric and biochemical data, respectively. In this calculation, height and WC are measured in centimeters (cm), while TG and HDL-C are expressed in milligrams per deciliter (mg/dL). The formula for calculating CMI is as follows:


$$\begin{array}{c} CMI=TG\left(mg/dL\right)/HDL-C\;\;\;\left(mg/dL\right)\times WHtR \end{array}$$ (1)

$$\begin{array}{c} \begin{array}{c} WHtR=waist\;\;\;circumference\left(cm\right)/height\left(cm\right) \end{array} \end{array}$$ (2)

### 2.3. Assessment of sleep disorders

Sleep disorders were assessed using the Sleep Disorder Questionnaire, specifically by querying the NHANES question SLQ050: “Have you ever told a doctor or other health professional that you have a sleep disorder?” Individuals who responded “yes” were classified as having a sleep disorder for further analysis. Additionally, respondents who answered “often” to questions SLQ030, SLQ040, or SLQ120 were also categorized as having a sleep disorder in subsequent analyses. This approach has been previously validated and widely used in population-based epidemiological studies.^[17–19]^ The sleep questionnaire items in NHANES are adapted from validated instruments including the Functional Outcomes of Sleep Questionnaire^[20]^ and have been employed in numerous studies examining sleep health in representative U.S. samples.

### 2.4. Study variables

Potential factors influencing the association between CMI and sleep disorders were selected based on a review of prior literature and subsequently screened using R software. The following variables were included: age, gender, race, educational level, poverty income ratio (PIR), BMI, fasting plasma glucose, total cholesterol (TC), low-density lipoprotein cholesterol, smoking status, alcohol consumption, history of depression, stroke, diabetes, and hypertension. Race was categorized into 5 groups: Mexican American, other Hispanic, non-Hispanic Black, non-Hispanic White, and other races. Educational level was stratified into 5 categories: <9th grade, 9 to 11th grade, high school graduate/GED or equivalent, some college or AA degree, and college graduate or higher. PIR was divided into 3 levels: ≤1.50, 1.50 to 3.00, and >3.00. Based on responses to questions SLQ101, SLQ110, and SLQ120Q, alcohol consumption was classified into never drinkers, former drinkers, and current drinkers. Subsequently, never drinkers and former drinkers were combined into a nondrinker category, while current drinkers were designated as drinkers. Similar to alcohol consumption, individuals were categorized as smokers or nonsmokers based on questions SMQ020 and SMQ040. Depression was assessed using a self-administered questionnaire, with a score of ≥10 indicating the presence of depression. Hypertension was determined by an average systolic blood pressure ≥ 130 mm Hg, an average diastolic blood pressure ≥ 80 mm Hg, or the use of antihypertensive medication.^[21]^ Participants were identified as having diabetes if they met one or more of the following standards: glycated hemoglobin (HbA1c) levels > 6.5%, fasting blood glucose levels ≥ 7.00 mmol/L, random blood glucose levels ≥ 11.10 mmol/L, a 2-hour oral glucose tolerance test result ≥11.10 mmol/L, or the current use of diabetes medications or insulin.^[22]^ The detailed measurement procedures for all these variables can be publicly accessed at www.cdc.gov/nchs/nhanes/.

### 2.5. Statistical analysis

All analyses were conducted in accordance with the guidelines provided by the Centers for Disease Control, with all data being weighted to account for the complex multistage sampling design of NHANES. Continuous variables were summarized as mean ± standard deviation, while categorical variables were presented as percentages. Weighted multivariate logistic regression was employed to assess the impact of CMI on sleep disorders. Model 1 was unadjusted; Model 2 was adjusted for age, gender, and race; and Model 3 further adjusted for gender, age, race, educational level, ratio of family income to poverty (Family PIR), alcohol consumption, diabetes, depression, stroke, and smoking status. Subsequently, receiver operating characteristic (ROC) curves and the area under the curve were calculated to compare the performance of CMI in predicting sleep disorders across different models. Weighted restricted cubic spline (RCS) regression analysis with 3 knots placed at the 10th, 50th, and 90th percentiles of the CMI distribution was performed to determine the nonlinear relationship between CMI and sleep disorders. Stratified multivariable logistic regression models were used for subgroup analyses and interaction tests. To assess the stability of the association between CMI and sleep disorders across various subgroups, we conducted subgroup analyses and interaction analyses using stratified multivariable logistic regression models. Missing data were handled using multiple imputation methods. Statistical analyses were performed using R software (version 4.4.1) and EmpowerStats (version 4.2). A two-sided *P*-value of <.05 was considered statistically significant.

## 3. Results

### 3.1. Characteristics of the study population

Based on the inclusion and exclusion criteria, a total of 5817 participants were included in the study, representing approximately 102 million non-institutionalized U.S. residents after weighting. Of these participants, 50.3% were female, with a mean age of 48.56 ± 16.97 years. Non-Hispanic White individuals comprised the majority of the sample, accounting for 64.3%. The average CMI was 1.39 ± 1.13, and the weighted prevalence of sleep disorders was 31.5%. Participants with sleep disorders were more likely to be older, female, non-Hispanic White, smokers, and to have a history of stroke, hypertension, diabetes, and depression (all *P* < .05) compared to those without sleep disorders. Additionally, those with sleep disorders exhibited higher levels of CMI (1.54 ± 1.18 vs 1.32 ± 1.09), BMI (30.93 ± 7.70 vs 28.92 ± 6.71), TC (190.61 ± 42.21 vs 186.79 ± 40.60, mg/dL), and fasting plasma glucose (112.76 ± 34.65 vs 108.41 ± 31.10, mg/dL). However, no statistically significant differences were observed between participants with and without sleep disorders in terms of educational level, PIR, alcohol consumption, and low density lipoprotein cholesterol levels. Weighted baseline characteristics of all participants, stratified by sleep disorder status, are provided in Table 1. Unweighted sample sizes for each subgroup are presented in Supplemental Digital Content 1, Supplemental Digital Content 1.

**Table 1:** Weighted baseline characteristics of the study participants.

Characteristics	Overall	Sleep disorders		*P*-value
		Without	With	
Age (yr)	48.56 ± 16.97	47.10 ± 17.32	51.73 ± 15.71	<.001
BMI (kg/m^2^)	29.55 ± 7.10	28.92 ± 6.71	30.93 ± 7.70	<.001
Gender (%)				
Male	49.7	52.9	42.7	<.001
Female	50.3	47.1	57.3	
Race (%)				
Mexican American	8.5	10.1	5.2	<.001
Other Hispanic	6.8	7.0	6.4	
Non-Hispanic White	64.3	61.6	70.1	
Non-Hispanic Black	10.5	11.0	9.4	
Non-Hispanic Asian	5.7	6.8	3.2	
Other race	4.2	3.5	5.7	
Education level (%)				
<9th grade	4.5	4.9	3.6	.151
9–11th grade	7.7	8.0	7.2	
High school	24.6	24.7	24.4	
Some college	30.5	29.1	33.6	
College or above	32.7	33.4	31.2	
PIR (%)				
<1.5	23.4	23.1	24.2	.100
1.5–3.0	26.0	27.2	23.3	
>3.0	50.6	49.7	52.5	
Smoke (%)				
Yes	17.5	16.0	20.7	.004
No	82.5	84.0	79.3	
Alcohol use (%)				
Yes	75.6	75.4	75.8	.838
No	24.4	24.6	24.2	
Hypertension (%)				
Yes	36.0	34.3	39.6	.015
No	64.0	65.7	60.4	
Diabetes (%)				
Yes	16.4	14.0	21.5	<.001
No	83.6	86.0	78.5	
Depression (%)				
Yes	9.3	4.9	18.9	<.001
No	90.7	95.1	81.1	
Stroke (%)				
Yes	3.2	2.1	5.5	<.001
No	96.8	97.9	94.5	
FPG	109.78 ± 32.32	108.41 ± 31.10	112.76 ± 34.65	<.001
TC	187.99 ± 41.15	186.79 ± 40.60	190.61 ± 42.21	.022
LDL-C	111.41 ± 35.65	111.32 ± 35.39	111.61 ± 36.24	.836
CMI	1.39 ± 1.13	1.32 ± 1.09	1.54 ± 1.18	<.001

Mean ± SD for continuous variables: the *P* value was calculated by the weighted linear regression model. (%) for categorical variables: the *P* value was calculated by the weighted chi-square test.

BMI = body mass index, CMI = cardiometabolic index, Family PIR = ratio of family income to poverty, FPG = fasting plasma glucose, LDL-C = low density lipoprotein cholesterol, TC = total cholesterol.

### 3.2. Association between CMI and sleep disorders

Table 2 presents the results of the multivariate logistic regression analysis examining the relationship between CMI and sleep disorders. A consistent positive association between CMI and sleep disorders was observed, regardless of whether CMI was treated as a categorical or continuous variable and whether adjustments for covariates were made. After adjusting for relevant covariates, Model 3 demonstrated that each one-unit increase in CMI was associated with a 17% higher likelihood of having a sleep disorder (odds ratio [OR] = 1.17; 95% confidence interval [CI]: 1.06–1.29; *P* = .004). When CMI was categorized into quartiles (0.56, 1.01, 1.90), participants in the highest quartile had an 81% increased risk of sleep disorders compared to those in the lowest quartile (OR = 1.81; 95% CI: 1.29–2.54; *P* = .002). Furthermore, the trend *P*-values for all 3 models were statistically significant (*P* < .05), indicating a meaningful trend across the quartiles.

**Table 2:** Associations between cardiometabolic index and sleep disorders.

Exposure	OR (95% CI)		
	Model 1	Model 2	Model 3
	(N = 5817)	(N = 5817)	(N = 5817)
CMI	1.18 (1.09, 1.29)*	1.21 (1.10, 1.33)*	1.17 (1.06, 1.29)**
CMI quartile			
Quartile 1 (<0.56)	Reference	Reference	Reference
Quartile 2 (0.56–1.01)	1.15 (0.90, 1.46)	1.15 (0.89, 1.48)	1.18 (0.91, 1.54)
Quartile 3 (1.02–1.90)	1.48 (1.11, 1.96)**	1.49 (1.11, 2.01)***	1.48 (1.09, 2.01)***
Quartile 4 (>1.90)	1.84 (1.36, 2.47)*	1.95 (1.40, 2.71)*	1.81 (1.29, 2.54)**
*P* for trend	<.001	<.001	<.001

In sensitivity analysis, CMI was converted from a continuous variable to a categorical variable (quartile).

Model 1 = No covariates were adjusted; Model 2 = Gender, age and race were adjusted; Model 3 = Gender, age, race, education level, Family PIR, drink, hypertension, diabetes, depression, fasting glucose, stroke and smoking status were adjusted.

95% CI = 95% confidence interval, CMI = cardiometabolic index, Family PIR = ratio of family income to poverty, OR = odds ratio.

* *P* < .001.

** *P* < .01.

*** *P* < .05, a *P* < .05 was considered statistically significant.

**Figure 2.**
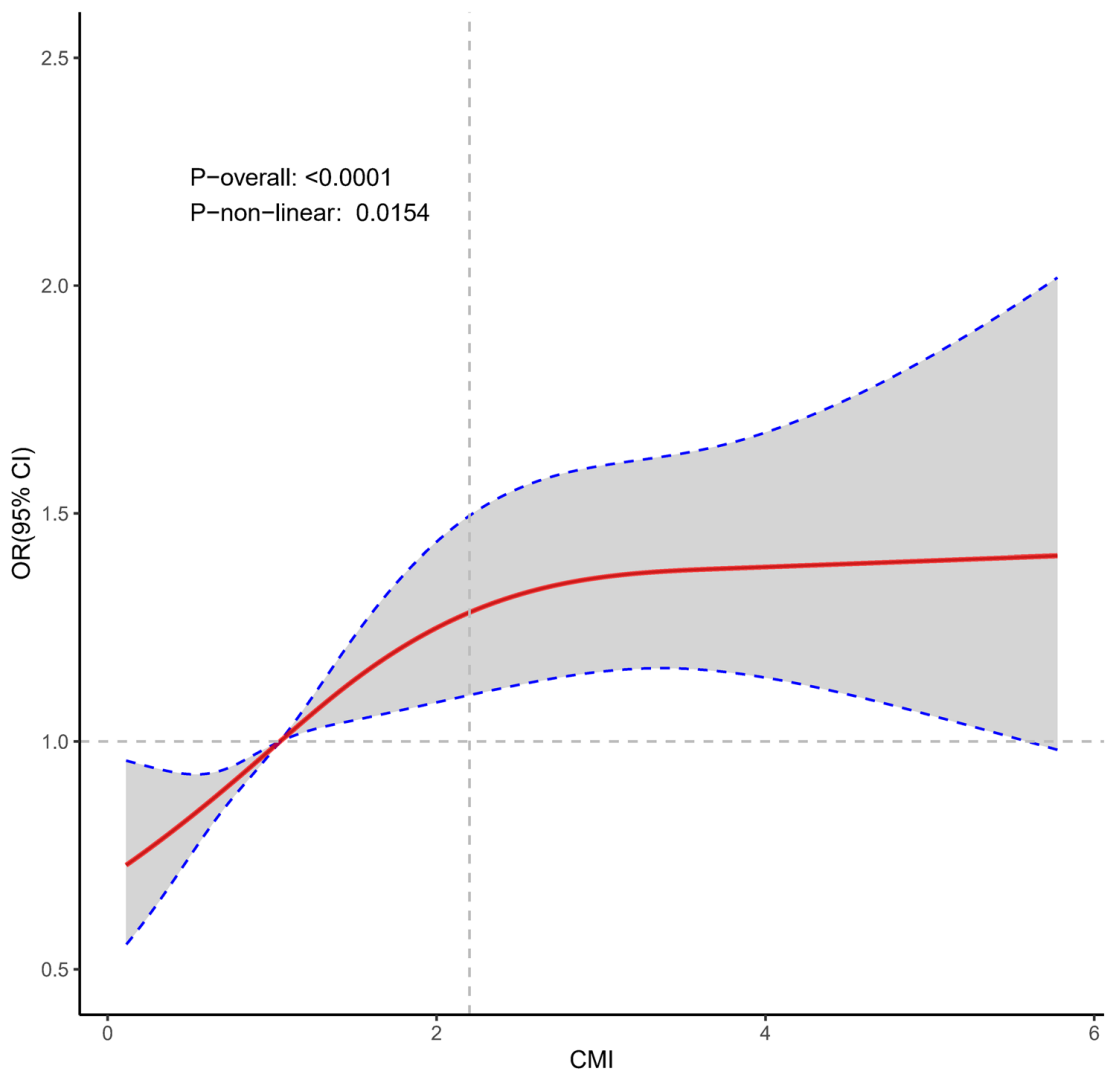
Determination of the association between CMI and sleep disorders by restricted cubic spline (RCS) regression analysis. CMI = cardiometabolic index, OR = odds ratio.

Additionally, in the RCS regression adjusted for potential covariates, a nonlinear positive association between CMI and sleep disorders was detected. As illustrated in Figure 2, when the CMI value was below 2.2, the risk of sleep disorders increased rapidly as CMI rose. However, once the CMI exceeded this threshold, the increase in the risk of sleep disorders began to plateau.

### 3.3. Receiver operating characteristic of CMI and sleep disorders

**Figure 3.**
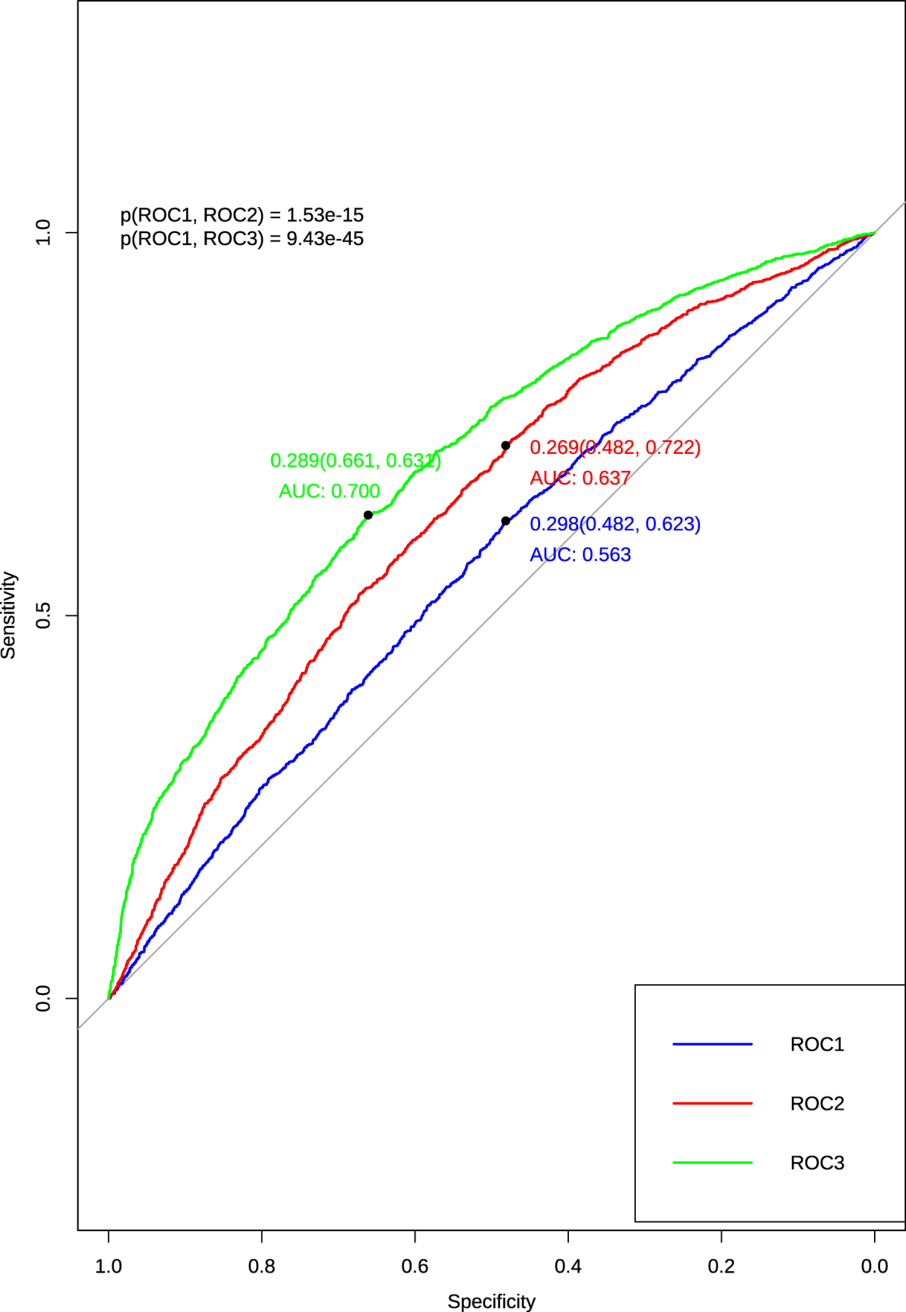
Receiver operating characteristic (ROC) curve for CMI and sleep disorders. ROC 1: unadjusted. ROC 2: adjusted for gender, age, and race. ROC 3: adjusted for gender, age, race, diabetes, and FPG. AUC = area under the curve, CMI = cardiometabolic index, ROC = receiver operating characteristic.

To evaluate the predictive efficiency of CMI for the risk of sleep disorders, weighted ROC curves were plotted (Fig. 3). All 3 models demonstrated a certain predictive ability, with the predictive power gradually improving as additional factors were adjusted. After adjusting for gender, age, race, educational level, Family PIR, alcohol consumption, diabetes, depression, stroke, and smoking status, the area under the curve for ROC3 reached 0.700. However, the specificity and sensitivity of the model still require further improvement.

### 3.4. Subgroup analysis

**Figure 4.**
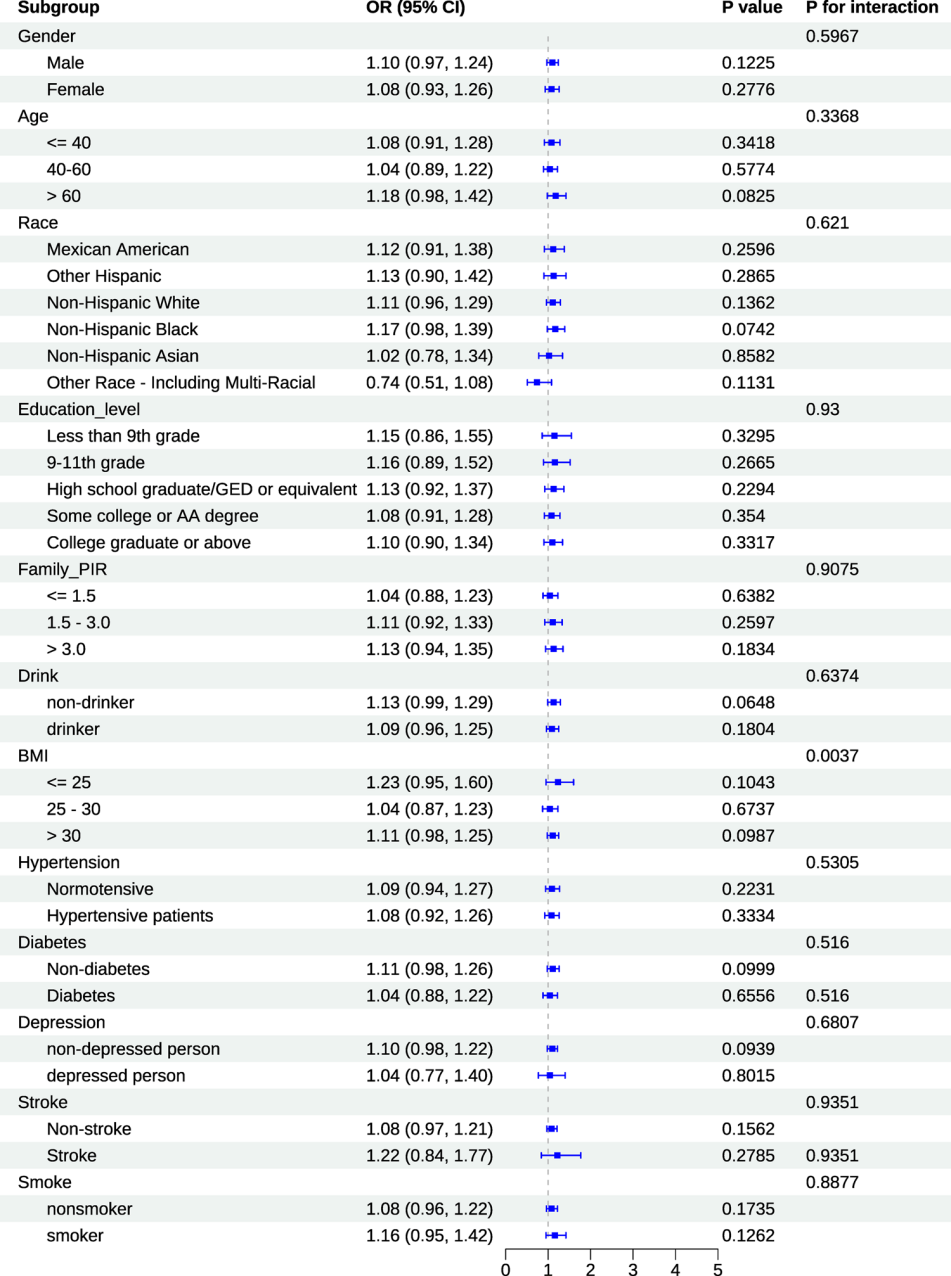
Verification of the association between CMI and sleep disorders by subgroup analyses. BMI = body mass index, Family PIR = ratio of family income to poverty, OR = odds ratio.

A subgroup analysis was conducted to assess whether the relationship between CMI and sleep disorders remained consistent across different population contexts. The results, presented in Figure 4, indicate that the association between CMI and sleep disorders was not significant at the subgroup level. Interaction tests revealed that gender, age, race, educational level, Family PIR, smoking, alcohol consumption, hypertension, diabetes, depression, and stroke did not significantly influence the positive association between CMI and sleep disorders (all *P* > .05). However, BMI was found to significantly impact the association between CMI and sleep disorders (*P* < .05).

## 4. Discussion

The objective of this study was to assess the relationship between CMI and sleep disorders. Our cross-sectional research, encompassing 5817 participants, revealed a significant increase in the likelihood of experiencing sleep disturbances among individuals with elevated CMI levels. Subgroup and interaction analyses demonstrated that this association remained consistent across various populations, with no other covariates significantly modifying this association except for BMI.

To the best of our knowledge, this is the first cross-sectional study to evaluate the association between CMI and sleep disorders. CMI, a newly developed lipid index, has been explored in various fields, particularly in studies concerning its correlation with diabetes and cardiovascular diseases among individuals with metabolically obese normal weight phenotypes.^[14,23–25]^ Our findings demonstrated a positive association between CMI and sleep disorders using multivariate logistic regression models, which further confirms the robustness of CMI in predicting lipid metabolism-related outcomes. Additionally, diabetes (OR: 1.40; 95% CI: 1.09–1.81) was found to significantly influence the risk of sleep disorders, underscoring the validity of the model. In interaction analysis, BMI (*P* = .0037) was shown to influence the predictive power of CMI, which is in line with expectations since BMI is a widely used international standard for assessing body fat and overall health. Furthermore, we found that female participants had a 14% higher risk of sleep disorders than male participants, which may be due to differences in hormone levels. Li et al found a positive correlation between estrogen metabolite levels and sleep efficiency, suggesting that higher estrogen levels are associated with better sleep quality.^[26]^ In addition, disrupted sex hormone levels have been associated with adipose tissue dysfunction and metabolic disorders in both sexes.^[27]^ Although we suspect a positive correlation between lipid metabolism and sleep disorders, the cross-sectional nature of this study does not allow us to establish a causal relationship. Numerous studies have investigated the effects of sleep disorders on fat metabolism, but there is now substantial research demonstrating the impact of fat metabolism on sleep disorders, indicating a bidirectional relationship between the 2. Rasaei N et al found that the cholesterol/saturated fatty acid index (CSI) affects sleep quality and circadian rhythms in a cross-sectional study of 378 obese or overweight adult women.^[28]^ Wang et al. showed that visceral adipose tissue may stimulate the release of inflammatory cytokines, which can disrupt sleep-wake patterns.^[29]^ Kuula L et al found that enhanced sleep quality was linked to increased TC levels, whereas shorter sleep duration was linked to decreased lipid levels.^[30]^ Taheri S et al also found that increased BMI led to shorter sleep duration in a longitudinal, population-based study of sleep disorders.^[31]^ Broussard J et al pointed out that adipose tissue may be a potential direct target of disrupting sleep quality.^[32]^ In addition, Dashti HS et al showed a consistent association between shorter sleep duration and higher total fat intake.^[33]^ Collectively, these findings support the utility of CMI as an integrative marker for sleep disturbances, consistent with the results of the present study.

Despite some studies exploring the potential mechanisms behind the positive correlation between CMI and sleep disturbances, the exact mechanisms of their interaction are still not fully understood. Romero-Corral A et al found that it may be related to fat deposition,^[34]^ as fat deposition around the upper airway tissues appears to lead to smaller and more collapsible upper airway lumens, making it more prone to sleep apnea. In addition, fat deposition around the chest (trunk obesity) decreases chest compliance and functional residual capacity and may increase oxygen demand, thereby leading to sleep disorders. Beyond its respiratory effects, android obesity has also been implicated in subclinical myocardial dysfunction. Speckle-tracking echocardiography studies have revealed that individuals with central obesity exhibit early impairment in left ventricular global longitudinal strain, independent of traditional cardiovascular risk factors.^[35]^ Moreover, recent evidence suggests that visceral adiposity is associated with subtle alterations in cardiac mechanics, which may precede the development of overt heart failure.^[36]^ These findings highlight the systemic cardiometabolic burden of android obesity, which may contribute to sleep disorders not only through mechanical respiratory impairment but also via shared inflammatory and autonomic pathways that affect both cardiac function and sleep regulation.

From a mechanistic standpoint, elevated CMI – reflecting visceral adiposity and insulin resistance – may promote a pro-inflammatory state characterized by increased levels of cytokinessuch as interleukin-6 (IL-6) and tumor necrosis factor-alpha (TNF-α). These mediators have been shown to penetrate the blood-brain barrier and interfere with key sleep-regulatory centers, including the hypothalamus, thereby disrupting circadian rhythms and sleep architecture.^[37]^ Conversely, sleep disorders can activate the sympathetic nervous system and the hypothalamic-pituitary-adrenal axis, leading to elevated cortisol and further exacerbating insulin resistance and adipose tissue dysfunction.^[38]^ This bidirectional interplay suggests a potential vicious cycle, wherein CMI and sleep disorders reinforce each other over time. Given the cross-sectional design of the present study, reverse causality cannot be ruled out; longitudinal studies are warranted to disentangle the temporal direction of this association. This broader perspective supports the potential role of CMI as an integrative marker linking adiposity, lipid metabolism, and sleep health. The intertwined nature of obesity and sleep is further reinforced by genetic evidence. Another significant discovery was that the single nucleotide polymorphisms associated with obesity and sleep are located on shared genes,^[29]^ which suggests that their relationship is not simply one of determination and being determined. Broussard J et al also hold a similar view, suggesting that the relationship between sleep disorders and lipid metabolism is like the chicken-and-egg dilemma.^[32]^

The primary strength of this study lies in its pioneering exploration of the relationship between CMI and sleep disorders, offering a novel perspective on the connection between lipid metabolism and sleep health. Additionally, potential confounding factors that could affect CMI were appropriately adjusted. The study further benefits from the use of a large, nationally representative sample of U.S. adults, enhancing the reliability and accuracy of the findings. However, the study also has certain limitations. First, due to the retrospective cross-sectional design and the reliance on preexisting NHANES data, we cannot establish a causal relationship between CMI and sleep disorders, nor can we infer the directionality of the observed associations. Moreover, sleep disorders were ascertained via self-reported questionnaires (e.g., SLQ050). The reliance on subjective recall may introduce nondifferential misclassification, potentially biasing the observed associations either toward or away from the null hypothesis. Second, despite adjusting for multiple covariates, the limitations of the NHANES database precluded us from including all potential confounders that may influence sleep disorders and lipid levels. This trade-off was necessary to maintain an adequate sample size but may introduce residual confounding.

## 5. Conclusion

In conclusion, our study suggests that elevated CMI levels are associated with an increased likelihood of sleep disorders, indicating that lipid metabolism management, as assessed by CMI, may help alleviate sleep disorders. However, further large-scale prospective studies are needed to validate these findings.

## Acknowledgments

We would like to convey our heartfelt appreciation to all those involved in this study.

## Author contributions

**Conceptualization:** Ximeng Peng, Yunyi Liu, Dong Wang.

**Data curation:** Ximeng Peng, Wenbo Wang, Congwenjun Zhang, Dong Wang.

**Formal analysis:** Ximeng Peng, Dong Wang.

**Methodology:** Ximeng Peng, Yunyi Liu, Dong Wang.

**Project administration:** Yunyi Liu.

**Software:** Ximeng Peng, Dong Wang.

**Supervision:** Yunyi Liu.

**Validation:** Wenbo Wang, Congwenjun Zhang.

**Visualization:** Ximeng Peng, Dong Wang.

**Writing – original draft:** Ximeng Peng.

**Writing – review & editing:** Ximeng Peng, Hengyue Liu, Dong Wang.


