# Supplementary material for: Associations between cardiometabolic index and sleep disorders: Results from NHANES 2015 to 2020
Source: Medicine (Baltimore). 2026 Jul 3;105(27):e49295. doi: 10.1097/MD.0000000000049295 (PMC13336968; doi:10.1097/MD.0000000000049295)
Supplement: Supplementary file 1 [file medi-105-e49295-s001.docx]

**Table S1** Unweighted baseline characteristics of the study participants.

| **Characteristics** | **ALL** | **No sleep disorders** | **Sleep disorders** | **p-value** |
| --- | --- | --- | --- | --- |
|  | ***N=5817*** | ***N=4122*** | ***N=1695*** |  |
| Gender: |  |  |  | <0.001 |
| Male | 2870 (49.3%) | 2138 (51.9%) | 732 (43.2%) |  |
| Female | 2947 (50.7%) | 1984 (48.1%) | 963 (56.8%) |  |
| Age | 50.7 (17.2) | 49.4 (17.6) | 53.7 (15.7) | <0.001 |
| Race: |  |  |  | <0.001 |
| Mexican American | 821 (14.1%) | 643 (15.6%) | 178 (10.5%) |  |
| Other Hispanic | 686 (11.8%) | 470 (11.4%) | 216 (12.7%) |  |
| Non-Hispanic White | 1989 (34.2%) | 1302 (31.6%) | 687 (40.5%) |  |
| Non-Hispanic Black | 1346 (23.1%) | 955 (23.2%) | 391 (23.1%) |  |
| Non-Hispanic Asian | 710 (12.2%) | 587 (14.2%) | 123 (7.26%) |  |
| Other Race - Including Multi-Racial | 265 (4.56%) | 165 (4.00%) | 100 (5.90%) |  |
| Education_level: |  |  |  | <0.001 |
| Less than 9th grade | 526 (9.04%) | 390 (9.46%) | 136 (8.02%) |  |
| 9-11th grade | 662 (11.4%) | 472 (11.5%) | 190 (11.2%) |  |
| High school graduate/GED or equivalent | 1349 (23.2%) | 982 (23.8%) | 367 (21.7%) |  |
| Some college or AA degree | 1813 (31.2%) | 1215 (29.5%) | 598 (35.3%) |  |
| College graduate or above | 1467 (25.2%) | 1063 (25.8%) | 404 (23.8%) |  |
| Family_PIR: |  |  |  | 0.040 |
| <= 1.5 | 1997 (34.3%) | 1389 (33.7%) | 608 (35.9%) |  |
| 1.5 - 3.0 | 1703 (29.3%) | 1246 (30.2%) | 457 (27.0%) |  |
| > 3.0 | 2117 (36.4%) | 1487 (36.1%) | 630 (37.2%) |  |
| Drink: |  |  |  | 0.862 |
| non-drinker | 1811 (31.1%) | 1280 (31.1%) | 531 (31.3%) |  |
| drinker | 4006 (68.9%) | 2842 (68.9%) | 1164 (68.7%) |  |
| BMI | 29.7 (7.17) | 29.0 (6.78) | 31.3 (7.81) | <0.001 |
| Hypertension: |  |  |  | <0.001 |
| Normotensive | 3386 (58.2%) | 2468 (59.9%) | 918 (54.2%) |  |
| Hypertensive patients | 2431 (41.8%) | 1654 (40.1%) | 777 (45.8%) |  |
| Diabetes: |  |  |  | <0.001 |
| Non-diabetes | 4531 (77.9%) | 3327 (80.7%) | 1204 (71.0%) |  |
| Diabetes | 1286 (22.1%) | 795 (19.3%) | 491 (29.0%) |  |
| Depression: |  |  |  | <0.001 |
| non-depressed person | 5185 (89.1%) | 3890 (94.4%) | 1295 (76.4%) |  |
| depressed person | 632 (10.9%) | 232 (5.63%) | 400 (23.6%) |  |
| FPG | 113 (38.5) | 112 (37.0) | 117 (41.8) | <0.001 |
| Stroke: |  |  |  | <0.001 |
| Non-stroke | 5562 (95.6%) | 3983 (96.6%) | 1579 (93.2%) |  |
| Stroke | 255 (4.38%) | 139 (3.37%) | 116 (6.84%) |  |
| Smoke: |  |  |  | 0.009 |
| nonsmoker | 4705 (80.9%) | 3370 (81.8%) | 1335 (78.8%) |  |
| smoker | 1112 (19.1%) | 752 (18.2%) | 360 (21.2%) |  |
| TC | 186 (41.4) | 186 (40.6) | 187 (43.5) | 0.212 |
| LDL_C | 110 (36.0) | 111 (35.3) | 110 (37.8) | 0.410 |
| CMI | 1.39 (1.11) | 1.33 (1.08) | 1.54 (1.16) | <0.001 |

Mean±SD for continuous variables: the P value was calculated by the weighted linear regression model. (%) for categorical variables: the P value was calculated by the weighted chi-square test.

Abbreviations: CMI, cardiometabolic index; BMI, body mass index; FPG, fasting plasma glucose; TC, total cholesterol; LDL-C, low density lipoprotein cholesterol; Family PIR, ratio of family income to poverty.
